# Familial adenomatous polyposis: ileo-anal pouch versus ileo-rectal anastomosis 

**Published:** 2014

**Authors:** Mohammad Mozafar, Kamran Shateri, Ali Tabatabaey, Saran Lotfollahzadeh, Khashayar Atqiaee

**Affiliations:** 1 *Department of General Surgery, Shahid Beheshti University of Medical Sciences, Tehran, Iran*; 2 *Department of Internal Medicine****, ****Urmia University of Medical Sciences, Urmia, Iran*; 3 *Department of Emergency Medicine, Shahid Beheshti University of Medical Sciences, Tehran, Iran*

**Keywords:** Familial adenomatous polyposis (FAP), J-pouch, Proctocolectomy

## Abstract

**Aim: **In this study we describe the presentation, treatment, and complications of 27 FAP patients.

**Background:** Treatment of Familial adenomatous polyposis (FAP) is centered on early recognition and curative surgery with either restorative proctocolectomy with ileal-pouch-anal-anastomosis (IPAA) or colectomy with ileo-rectal anastomosis (IRA).

**Patients and methods: **All patients diagnosed with FAP at our center from 2008 to 2012 were included in this case series. Either IPAA or IRA was used for treatment. Complications were recorded for 12 months after the procedure.

**Results:** Overall 27 patients were included, 12 (44.44%) index patients, and 15 (55.55%) relatives diagnosed by screening. Eight Index patients presented with rectal bleeding, two with occult fecal blood and two with abdominal masses found to be desmoid tumors. Nineteen patients were treated by IPAA, 6 with IRA, and 2 were inoperable due to diffuse desmoid tumors. Daytime stool frequency was the most common side effect (70.37%), followed by bowel discomfort episodes (55.56%), requiring dietary restrictions (37.4%), passive incontinence (25.93%), soiling (22.22%), nighttime stool frequency (18.52%), flatus incontinence (16.0%), and anastomosis leakage (3.70%). On average patients treated by IPAA experienced less complication than those treated by IRA.

**Conclusion:** compared with previous reports, this series had older age of diagnosis, higher rate of adenocarcinoma at diagnosis, and fewer side effects after IPAA than IRA. The latter may reflect technique improvement with experience, and if supported by future studies, will cement IPAA as the treatment of choice in FAP.

## Introduction

 Familial adenomatous polyposis (FAP) is a disorder characterized by the early onset of hundreds to thousands of adenomatous polyps throughout the colon. It is the most common adenomatous polyposis syndrome with an average age of onset of 16 years. Disease frequency is thought to be constant, regardless of geographic location, sex, or race (1) and all untreated patients go on to develop colon cancer by the age of 35-40 years. FAP is caused by a germline mutation in the adenomatous polyposis coli (APC) gene on chromosome 5, which is inherited in an autosomal dominant fashion (2,3).

Historically treatment options for both prophylactic and curative surgery include proctocolectomy and terminal ileostomy (PCI), total colectomy and ileorectal anastomosis (IRA) and restorative proctocolectomy with ileal pouch anal anastomosis (IPAA). Due to the tendency of the disease to turn malignant, procedures that remove the whole colon and rectum are favored. But PCI is associated with significant body image alteration. Thus, total proctocolectomy was proposed early on (4) to combine the prophylactic nature of removing the whole colon with a better body image by saving the rectal sphincter. A major drawback was significant complications affecting quality of life. The technique was reintroduced in the late 70s (5) and this time modifications were made to lessen the side effects and improve quality of life (,). These improvements have led to the virtual elimination of PCI as a prophylactic measure, leaving either IRA or IPAA for this purpose (10-12).

In this study we aim to describe the clinical presentation, demographics, and short-term complications of the patients treated for FAP at our center. 

## Patients and Methods

This was a case series of patients treated for FAP at Shohada Tajrish Medical Center from fall 2008 to fall 2012. Three groups of patients are included in this study. The first group is patients referred to the center for definitive treatment with a previously established diagnosis of FAP. The second group is those patients who presented to the emergency department at Shohada Tajrish and were diagnosed with FAP at our center. The third group is family members of index patients who were diagnosed through screening investigations. During the study period every patient diagnosed with FAP was registered and demographic information was gathered alongside specific information on clinical signs and symptoms. All patients underwent upper and lower gastrointestinal endoscopy. 

Based on physician and patient preference and disease status one of two treatment options were carried out. IRA was presented as an option only in cases where the rectal mucosa was found to be uninvolved on proctoscopic examination. IPAA was performed as a two-step procedure. The first step involved proctocolectomy followed by the creation of an ileal pouch, and anastomosis of this pouch to the remaining anus using an end-to-end anastomosis stapler between the pouch and the rectal cuff. In order to create the pouch ileum was folded on itself in the shape of a J, and the two bowel segments were connected. In this step a diverting ileostomy was placed. The ileostomy was closed 6 weeks later in the second step of the procedure, and the two bowel loops were connected using GIA™ stapler. Alternatively IRA was performed using total colectomy and primary ileo-rectal anastomosis.

Family members of patients at risk were also called in for diagnostic evaluation and in cases with a positive diagnosis, the same protocol was followed as with index patients and treatment was implemented. 

All patients were followed up at least 12 months after treatment and early complications were recorded. Special focus was put on known side effects such as anastomosis stricture, nighttime and daytime stool frequency, anastomosis leakage, bowel discomfort episodes, and fistula formation. Descriptive data analysis was conducted using IBM-SPSS software version 21. 

## Results

Overall 27 patients suffering from FAP were treated at our center during the study period. Of these, 12 (44.44%) were index patients presenting with either rectal bleeding (n=8) or occult fecal blood (n=2). Two patients presented with abdominal masses and a diagnosis of desmoid tumors, complicating an undiagnosed or neglected FAP. The rest of the patients (55.55%) were family members of the index patients recognized via colonoscopy screening. Mean age of presentations was 37.15 (range 21 to 50) and the majority of patients were male (n=21, 77.77%). Table 1 summarizes the study results.

**Table 1:** Summary of findings

Variables		%
Gender		
	Male	77.8
	Female	22.2
Signs& Symptoms on presentation		
	Screening	55.5
	Rectal bleeding	29.6
	Weight loss	14.8
	Occult blood	7.4
	Weakness and anemia	3.7
Endoscopy results		
	Adenomatous polyps	66.6
	Chronic duodenitis on endoscopy	18.5
	Adenomatous polyps with dysplasia	14.8
	Poorly differentiated adenocarcinoma	11.1
	Moderately differentiated adenocarcinoma	7.4
Treatment		
	Daytime stool frequency	70.4
	Bowel discomfort episodes	55.5
	Dietary restrictions	37.04
	Anti-diarrheal medication	29.6
	Passive incontinence	25.9
	Soiling	22.2
	Nighttime stool frequency	18.5
	Flatus incontinence	14.8
	Anastomosis leakage	3.7

At the time of diagnosis moderately and poorly differentiated adenocarcinoma was detected in 2 (7.40%) and 3 (11.11%) patients respectively, all of whom were diagnosed by screening. Paraneoplastic findings were also sought in all patients. Alongside two patients with desmoid tumors (7.40%), one patient was diagnosed with mandibular osteomas (3.70%), and a fourth patient was found to have juvenile nasopharyngeal angiofibromas plus congenital hypertrophy of the retinal pigment epithelium (3.70%).

Nineteen patients were treated by IPAA, 6 with IRA, and 2 were inoperable due to a diagnosis of desmoid tumor. During the one-year post-op follow up, none of the patients displayed complications of perianal irritation, anastomosis stricture, small bowel obstruction, fistula formation, or mesenteric venous thrombosis. Daytime stool frequency was the most common side effect of treatment experienced (70.37%), followed by bowel discomfort episodes (55.56%). Ten patients required dietary restrictions (37.4%), while 8 required administration of anti-diarrheal medication (29.63%). Passive incontinence was reported in 7 patients (25.93%), soiling in 6 (22.22%), nighttime stool frequency in 5 (18.52%), flatus incontinence in 4 (16.0%), and anastomosis leakage in only one patient (3.70%). 

On average patients treated by IPAA experienced fewer complications per person (2.73, median=3) when compared to the group treated by IRA (3.66, median=4). Table 2 summarized the percentage of patients affected by complications in each treatment group. 

**Table 2:** Rate of complications in each treatment group

Complication	IPAA	IRA
Anastomosis leakage	0.0%	16.7%
Antidiarrheal meds	26.3%	50.0%
Bowel discomfort	57.9%	66.7%
Daytime frequency	78.9%	66.7%
Dietary restrictions	36.8%	50.0%
Flatus incontinence	10.5%	33.3%
Nighttime frequency	21.1%	16.7%
Passive incontinence	21.1%	50.0%
Soiling	26.3%	16.7%

## Discussion

In this study we describe the demographic information, clinical manifestation, and treatment complications of 27 patients with FAP at Shohada Tajrish Medical Center between fall of 2008 and 2012. The mean age of diagnosis in our study was over 37 years, which is significantly higher than the age reported in the literature (2) . This is alarming since by the time patients reach their mid-thirties, colon cancer starts to develop. This is, to some extent, reflected by the fact that nearly 20% of our patients had already developed moderate or poorly differentiated carcinoma at the time of diagnosis. Furthermore, 7.40% were suffering from inoperable desmoid tumors, a paraneoplastic syndrome associated with FAP. Desmoid tumors or diffuse mesenteric fibromatosis are the primary cause of death after prophylactic surgery has been performed for FAP (11-13). This diffuse intra-abdominal mass is reported in 4-32% of patients and the mortality for these tumors ranges between 10-50% (1,14). Many of our index patients had been experiencing symptoms for years without a definite diagnosis. This signals the need for a higher alertness on the part of the primary care physicians for the diagnosis of FAP. 

The second most common malignancy in patients with FAP is adenocarcinoma of the duodenum and the ampulla of Vater, which affects up to 12% of patients (1). In our study none of the patients were found to have upper GI malignancy while duodenitis was confirmed in 5 patients. 

Currently two treatment options are available for FAP, IRA and IPAA. A meta-analysis of over 1000 patients found that each option has its benefits and downfalls. While bowel frequency, night defecation and use of incontinence pads were significantly less in the IRA group, fecal urgency was reduced with IPAA (15). Another study looking at 323 patients treated for FAP also found that functional results including daytime and nighttime stool frequency, soiling, occasional passive incontinence, flatus and feces discrimination, stool consistency, and need for antidiarrheal medication, was significantly better for patients undergoing IRA (11). It has also been suggested that age might affect the frequency of side effects in IPAA (16). Yet the greatest advantage offered by IPAA is the elimination of colorectal cancer development. This is an advantage that has made IPAA the treatment of choice for FAP in many centers. Furthermore it's been put forth that greater experience with performing IPAA can decrease the rate of side effects while preserving its curative nature (17). In our study, although the small number of patients did not allow concrete statistical analysis, we found that the average number of complications reported was lower in patients undergoing IPAA (3.66 compared with 2.73). Moreover, we found that except for daytime and nighttime frequency and occasional soiling, all other complications were reported with greater frequency in the group treated with IRA. This might be in part due to the fact that IPAA was performed with a diverting ileostomy while IRA was achieved by primary anastomosis. Also it might be because greater experience with IPAA has improved its side effects profile to a level comparable with IRA.

In the series of patients described here, older age of diagnosis for patients translated into a higher rate of adenocarcinoma at the time of diagnosis. Furthermore patients reported fewer side effects within the first year after IPAA as compared to IRA. Although statistical significance was not measurable due to the limited number of patients in the study, we believe that this might be a reflection of technique improvement. If so it can be expected that with further experience and the introduction of minimally invasive methods of performing IPAA (18), it is bound to gain further support as the treatment of choice in patients suffering from FAP.
